# Two Co(II) coordination polymers: magnetic properties and application values against chronic subdural hematoma

**DOI:** 10.1080/15685551.2021.1935535

**Published:** 2021-06-28

**Authors:** Jing-Sen Chen, Sheng-Long Cao, Hai-Ying Hu, Juan Shen, Yu-Jun Qi

**Affiliations:** a Department of Neurosurgery, The Second Affiliated Hospital, Medical School, Zhejiang University, Hangzhou, Zhejiang, China; b Department of Nutriology, The Second Affiliated Hospital, Medical School, Zhejiang University, Hangzhou, Zhejiang, China; c Department of Medicine, Nanjing Medical University, Huai’an, Jiangsu, China

**Keywords:** Coordination polymers, mixed-ligand, magnetic performances, chronic subdural hematoma

## Abstract

In this current experiment, by applying the mixed-ligand synthesis method, two coordination polymers (CPs) containing Co(II) were created triumphantly with reaction between 1,3-bis(1-imidazoly)benzene (mbib) and Co(II) salts with the aid of diverse carboxylic ligands, and their chemical formulae are [Co_3_(opda)_3_(mbib)_4_(H_2_O)_4_]·2H_2_O (**1**, H_2_opda is 1,2-phenylenediacetic acid) and [Co(mpda)(mbib)]·H_2_O (**2**, H_2_mpda is 1,3-phenylenediacetic acid). The two compounds’ magnetic performances suggest that between the adjacent metal ions, there present the antiferromagnetic coupling. The evaluation of their treatment activity against chronic subdural hematoma was carried out and the relevant mechanism was studied simultaneously. Firstly, before the treatment of compound, the chronic subdural hematoma was generated. Furthermore, the enzyme-linked immunosorbent assay detection kit was implemented and in hematoma capsule, the anti-inflammatory cytokines level and pro-inflammatory cytokines level was detected. Additionally, the cytotoxicity of compounds **1** and **2** on the normal human cells was determined with Cell Counting Kit-8 assay. Above all, we proved compound **1** decreased the pro-inflammatory cytokines content and increased the anti-inflammatory cytokines content in the hematoma capsule, which is much stronger than that of compound **2**. Both compounds **1** and **2** showed no cytotoxicity on the normal human cells.

## Introduction

Chronic subdural hematoma (CSDH) is one of the familiar diseases in neurosurgery, most of which are elderly patients. Drilling and drainage are currently the most effective method for the clinical treatment of CSDH, but the fear of surgery and postoperative recurrence in elderly patients has always plagued the clinic application [1]. Therefore, it is important to explore the pathogenesis of CSDH and explore effective non-invasive CSDH treatment methods. In the CSDH process, the imbalance of pro-inflammatory cytokines and dysfunctional anti-inflammatory is often accompanied [2,3]. Thus, new candidates were developed for the treatment of CSDH targeting anti-inflammatory and pro-inflammatory cytokines balance.

In the past several years, the investigation and establishment of the coordination polymers (CPs) have received major study hotspots in the area of materials science owing to their various structures of topology and they have broad application prospects in the gas storage, magnetism, luminescence, catalysis, molecular separation and sensing, ion exchange, biomedicine and dye adsorption fields [4–7]. In general, CPs are composed of metal centers and organic ligands, and their final structures are high dependent on lots of factors, for example, the metal ions, solvents, temperature of reaction, reagent ration and ligands, etc. [8–11]. Among these factors, the selection of suitable organic ligands possesses a major effect in the CPs’ ultimate structures via the effects of the ligand coordinative atoms along with the ligand geometry. Therefore, we chose the rigid ligand of 1,3-bis(1-imidazoly)benzene (mbib) and two diverse feasible carboxylic ligands to be utilized in the mixed-coordination system, on the basis of these characteristics: (i) the position of the two carboxyl groups is more conducive to the production of the uncoordinated O atoms with latent hydrogen-bond donor positions, (ii) when the 2 carboxyl groups of carboxylic acid ligands coordinated with the Co(II) ions, they could reveal more rich coordination manner, and (iii) the –CH_2_-free rotation can facilitate the dicarboxylic acid ligand flexibility to meet the coordination geometric requirements of the ultimate structure constructed by the Co(II) ions [12–17]. Meanwhile, the strong coordination ability of imidazole N atoms is prone to produce stable frameworks. Bearing these in mind, in the present experiment, by applying the mixed-ligand synthesis method, two fresh CPs containing Co(II) were generated triumphantly with reaction between 1,3-bis(1-imidazoly)benzene (mbib) and Co(II) salts with the aid of diverse carboxylic ligands, and their chemical formulae are [Co_3_(opda)_3_(mbib)_4_(H_2_O)_4_]·2H_2_O (**1**, H_2_opda is 1,2-phenylenediacetic acid) and [Co(mpda)(mbib)]·H_2_O (**2**, H_2_mpda is 1,3-phenylenediacetic acid). The two compounds’ magnetic performances suggest that between the adjacent metal ions, there present the antiferromagnetic coupling. Serial biological experiments were performed in the current work for the assessment of the compound application values against CSDH. The Cell Counting Kit-8 assay indicated that both compounds **1** and **2** showed no cytotoxicity on the normal human cells.

## Experimental

### Chemicals and measurements

All the chemicals applied for the production of the complexes could be acquired from the market, and these could be utilized without processing. For the infrared spectrum, it could be recorded employing the spectrometer of Nicolet 170SX as the KBr pellets, with spectrum region varying from 400 cm^–1^ to 4000 cm^–1^. The Hydrogen, Nitrogen and Carbon elements can be analyzed through analyzer of 2400 model Perkin-Elme. The XRPD could be recorded utilizing the Cu Kα radiation through applying the diffractometer of PANalytical X’Pert Pro. The TGA could be implemented between ambient temperature and 800°C with the thermogravimetric analyzer of Perkin-Elmer TGA-7 at 10°C·min^–1^ heating rate under the atmosphere of nitrogen. For all the constituent atoms, their antimagnetic corrections were implemented through employing Pascal’s constants.

### Preparation and characterization for [Co_3_(opda)_3_(mbib)_4_(H_2_O)_4_]·2H_2_O (1) and [Co(mpda)(mbib)]·H_2_O (2)

The mixture created by 0.0210 g and 0.1 mmol of H_2_opda, 0.1 mmol and 0.030 g of Co(NO_3_)_2_ · 6H_2_O, 0.0194 g and 0.1 mmol of mbib and 0.1 mmol and 5.6 mg of KOH were added into the 12 mL of water in a stainless steel vessel with Teflon lining (25 mL). This acquiring mixture was heated for seventy-two hours at 160°C, and then it was gradually cooled to the ambient temperature. In the end, the complex **1**’s pink massive crystals were gained. Elemental analysis calcd. (%) for the C_78_H_76_N_16_Co_3_O_18_: N, 13.16%; C, 55.03% and H, 4.50%; found (%): N, 13.11%; C, 54.99% and H, 4.58%. IR (KBr pellet, cm^−1^): 503 (w), 533 (w), 653 (m), 682 (m), 729 (m), 763 (m), 777 (w), 846 (w), 863 (w), 903 (w), 933 (m), 1014 (m), 1075 (m), 1107 (w), 1168 (m), 1241 (m), 1286 (w), 1303 (w), 1381 (m), 1448 (m), 1507 (s), 1538 (m), 1569 (m), 1606 (m), 1616 (s), 1685 (s), 2871 (w), 3134 (w), 3448 (w).

The mixture formed by 0.0210 g and 0.1 mmol of H_2_mpda, 0.1 mmol and 0.030 g of Co(NO_3_)_2_ · 6H_2_O, 0.0194 g and 0.1 mmol of mbib and 0.1 mmol and 5.6 mg of KOH were added into the 12 mL of water in a stainless steel vessel with Teflon lining (25 mL). This obtaining mixture was heated for seventy-two hours at 140°C, and then it was gradually cooled to the ambient temperature. As a result, the complex **2**’s pink massive crystals were acquired. Elemental analysis calcd. (%) for the C_22_H_20_N_4_CoO_5_: N, 11.69%; C, 55.12% and H, 4.21%; found (%): N 11.23%; C 54.92% and H, 4.27%. R (KBr pellet, cm^−1^): 535 (m), 648 (m), 703 (w), 770 (m), 823 (m), 936 (w), 960 (m), 1061 (m), 1129 (w), 1163 (w), 1236 (m), 1310 (m), 1364 (m), 1385 (m), 1446 (m), 1516 (s), 1558 (m), 1590 (m), 3116 (m), 3619 (w).

The X-ray data can be gained through applying the SuperNova diffractometer. For the analysis of the strength data, software of CrysAlisPro was utilized, and this data was then converted to files of HKL. The original patterns of structure can be generated via exploiting direct mean based program of SHELXS, and subsequently, the least-squares method based program of SHELXL-2014 was employed to modify. The anisotropic parameters were mixed with the overall non-hydrogen atoms. Ultimately, via applying AFIX commands, the overall hydrogen atoms were fixed on carbon atoms in geometry which they are attached. For the two complexes, their parameters of crystallography as well as the details of refinement are exhibited in length in Table 1.

**Table 1.** The compounds’ parameters of crystallography as well as the details of refinement

Identification code	1	2
Empirical formula	C_78_H_76_Co_3_N_16_O_18_	C_22_H_20_CoN_4_O_5_
Formula weight	1702.33	479.35
Temperature (K)	293.15	296.15
Crystal system	triclinic	orthorhombic
Space group	P-1	Pbcn
*a* (Å)	10.9732(2)	15.1529(11)
*b* (Å)	12.6658(3)	17.2633(14)
*c* (Å)	15.1425(2)	15.7425(9)
*α* (°)	103.369(5)	90
*β* (°)	108.5270(10)	90
*γ* (°)	95.639(3)	90
Volume (Å^3^)	1907.48(7)	4118.1(5)
*Z*	1	8
*ρ*_calc_ (g/cm^3^)	1.482	1.546
*μ* (mm^−1^)	0.727	0.877
Data/restraints/parameters	9695/3/579	5194/0/289
Goodness-of-fit on *F*^2^	1.197	1.068
Final *R* indexes [I ≥ 2*σ* (I)]	*R*_1_ = 0.0484, *ωR*_2_ = 0.1316	*R*_1_ = 0.0445, *ωR*_2_ = 0.0981
Final *R* indexes [all data]	*R*_1_ = 0.0543, *ωR*_2_ = 0.1340	*R*_1_ = 0.0818, *ωR*_2_ = 0.1152
Largest diff. peak/hole/e (Å^−3^)	1.13/-0.35	0.72/-0.23
CCDC	2,064,637	2,064,638

### CSDH animal model

For the assessment of the new compound’s biological activity against the treatment of CSDH, the CSDH animal model was established firstly in our work. This investigation was carried out completely in accordance with the instructions with a little modification. In short, forty Wistar rats (6–8 weeks, 200–220 g) applied in our study were offered by the Jinan University (Jinan, China), and these rates were separated randomly into the sham operation group, CSDH group, the compound **1** treatment and compound **2** treatment groups. Afterwards, intracranial subdural space was injected with autologous venous blood every two 3-day intervals, the injection of two compounds were carried out for the treatment (with 2.5 μg/kg concentration). PBS was given as negative control to the rats in the sham group.

### ELISA assay

After the production of animal model and the treatment of compounds, the enzyme-linked immunosorbent assay was performed and the anti-inflammatory cytokines content and pro-inflammatory cytokines content released from hematoma capsule was detected. This conduction is finished in the light of protocols with minor change. Briefly, in intracranial subdural space, the autologous venous blood was injected into the Wistar rats every two 3-day intervals, and after that, the two compounds were exploited to perform the treatment (at the concentration of 2.5 μg/kg) two hours before the modeling of CSDH; in sham group, the PBS was injected into rats as the identical way. Subsequently, the hematoma capsule could be harvested and the TNF-α content and IL-10 content was determined. This present research was implemented at least 3 times and the obtaining outcomes were reflected as the mean±SD.

### Cell Counting Kit-8 assay

The Cell Counting Kit-8 assay was conducted in this present research to determine the inhibitory activity of compounds **1** and **2** on the viability of the normal human cells. This preformation was carried out totally in accordance with the manufactures’ protocols with only a little change. In brief, the normal human cells in the logical growth phage were collected and seeded into the 96 well plates at the final destiny of 10^5^ cells/well. Then, the cell was cultured in an incubator at the condition of 37°C, 5% CO_2_ for 12 hours. Then, compounds **1** and **2** was added for treatment with serial different dilutions (1, 2, 4, 8, 10, 20, 40 and 80 μM). After 48 hours incubation, the cell culture medium was discarded and new medium containing CCK-8 reagent was added for further 4 hours incubation. Finally, the absorbance of each well was determined with Microplate reader at 490 mm. This experiment was repeated at least three times, and the results were presented as mean±SD.

## Results and discussion

### Crystal structures

**Figure 1.**
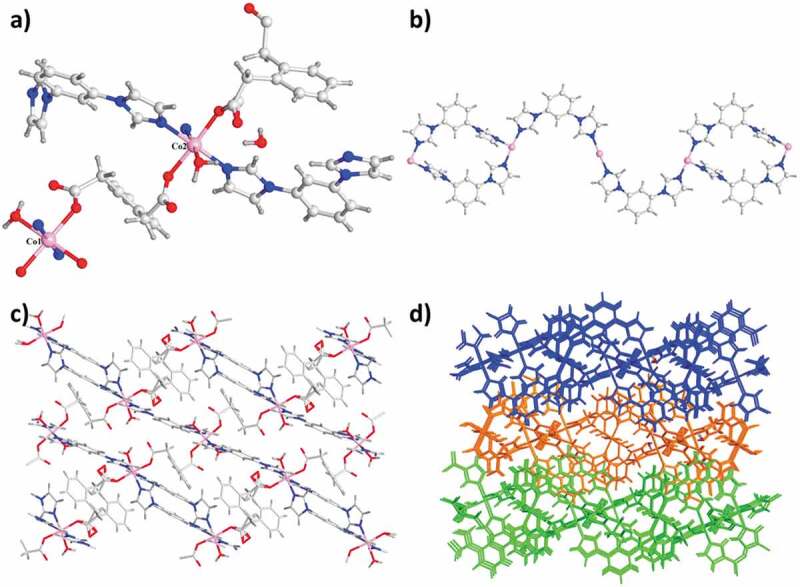
(a) The **1**’s asymmetry unit. (b) The one-dimensional loop of [Co_2_(mbib)_2_] in complex **1**. (c) The **1**’s two-dimensional layered net. (d) The three-dimensional packing diagram of the complex **1.**

The analysis for the diffraction of single-crystal X-ray indicated that in space group *P-1* of triclinic crystal system, complex **1** could crystallize. The Co1 atom is located in the distorted coordination geometry of octahedron and it was coordinated via 2 N atoms come from 2 distinct ligands of mbib and 4 O atoms in 2 diverse ligands of opda^2−^ and 2 molecules of water. While the Co2 atom also reveals six-coordinated having a distorted coordination sphere of octahedron, which is synthesized from 3 N atoms come from 3 distinct ligands of mbib and 3 O atoms originate from 2 diverse ligands of opda^2−^ and a molecule of water (Figure 1(a)). In the complex **1**, there exist two kinds of the ligands mbib exhibiting the non-plan conformation, between the imidazole arms and benzene core, the dihedral angle is 4.96° and 23.23°, and 8.32° and 69.64°, respectively. As a result, the two distinctive Co1 atoms are connected via two ligands of mbib to create a loop [(Co1)_2_(mbib)_2_]. The neighboring loops are in-depth linked via a motif of [Co_2_(mbib)_2_] to produce a one-dimensional loop having the polymer chains. OPDA in-depth extends these one-dimensional chains with the bis(monodentate) coordination fashion of μ_1_–η^1^:η^0^ to create a two-dimensional (4,4) network (Figure 1(b,c)). Moreover, the neighboring two-dimensional networks are in-depth interconnected to extend into a whole three-dimensional supramolecular net along axis *c* through these interactions: the hydrogen bond interactions of C–H⋯O between the opda^2−^ ligands carboxylic acid oxygen atoms and benzene rings (C45–H45⋯O10) and the interlayer C–H⋯π between benzene rings of the opad^2−^ ligand and mbibl ligand (C36–H36⋯π, and the separations of C⋯π is 3.373 Å) (Figure 1(d)).

**Figure 2.**
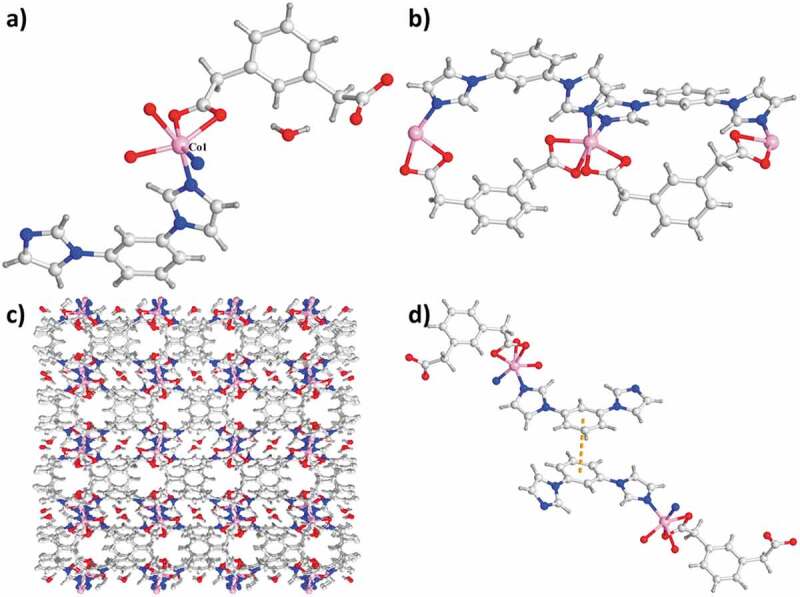
(a) The asymmetry unit of complex **2**. (b) The **2**’s one-dimensional loop chain architecture. (c) The three-dimensional packing network of complex **2**. (d) The interactions of π⋯π between the neighboring chains of the complex **2.**

The structure analysis of single crystal X-ray indicated that the complex **2**’s asymmetric unit is constructed from a totally deprotonated ligand of mpda^2−^, a Co(ii) atom, a lattice molecule of water and a ligand of mbib. As reflected in the Figure 2(a), each of the Co(ii) atom in complex **2** is six-coordinated via 4 oxygen atoms come from 2 distinct ligands of mpda^2−^ [the distance of Co1–O1, Co1–O2, Co1–O3A and Co1–O4A respectively are 2.154(2), 2.091(2), 2.083(2), and 2.039(2)Å] and 2 nitrogen-atom donors in 2 ligands of mbib [the length of Co1–N1 and Co1–N4A respectively are 2.023(2) and 2.039(2) Å]. In the complex **2**, between a benzene core and two imidazole arms, the dihedral angle respectively is 11.06° and 28.81°. The mpda two carboxylic groups tend to be on the same side of benzene ring plane via the chelating coordination manner and μ_1_–η^1^:η^1^ coordination manner. As a result, the ligand of mpda^2−^ also exhibits the *cis*-conformation and it can be regarded as the building block with ‘V’-type. The Co(ii) atoms are linked together to provide the one-dimensional loop involving the polymeric ribbons along the direction *a* with the mixture between the ppda^2−^ ligand, mbib ligand and two building blocks with ‘V’-type (Figure 2(b)). In the complex **2**, the Co⋯Co length through the ligand of mbib is 8.830 Å, which is shorter than the length of 10.904 Å in complex **1**. As displayed in the **2**’s crystal stacking illustration, the conservative one-dimensional polymer ribbon motifs are combined via the strong hydrogen bonds between molecular to produce a two-dimensional supramolecular layer. The H-bonding system in the complex **2** is composed of the H-bonding interactions of C–H⋯O between the mpda carboxylic acid oxygen atoms come from a mpda^2−^ ligand and the mpad^2−^ methylene from ribbon. Furthermore, the extended three-dimensional porous supramolecular net is generated between benzene rings of 2 diverse ligands of mbib having face-to-face orientation through the interactions of π⋯π (the distance between centroid and centroid is 3.647 Å) (Figure 2(c,d)). The lattice molecules of water can be fixed in channels through the H-bonding interactions of O–H⋯O (the distance of O5–H5B⋯O2 and O5–H5⋯O3 are 2.258 Å and 2.064 Å) and C–H⋯O (the lengths of C7⋯O5 and C4⋯O5 are 3.731 Å and 2.900 Å, respectively). Evidently, these strong interactions of H-bonding have a major effect in producing the three-dimensional supramolecular structures.

**Figure 3.**
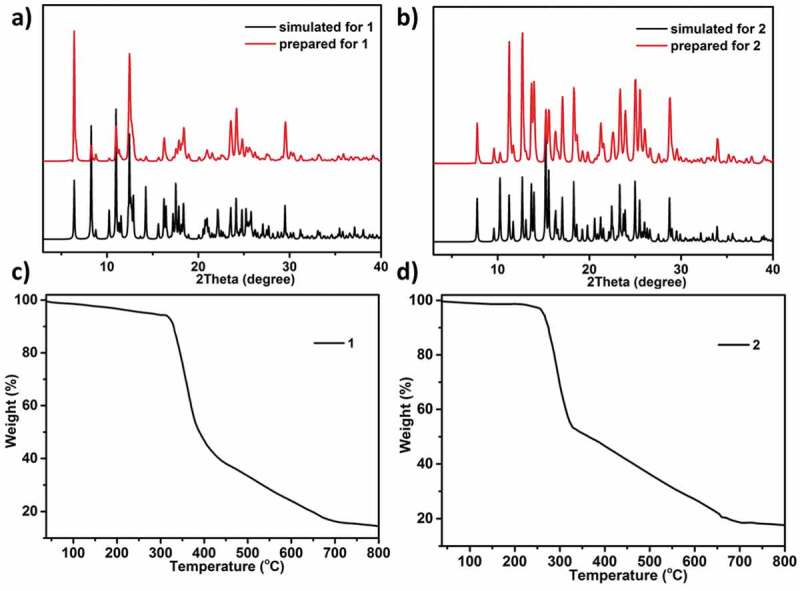
The PXRD models of complex **1** (a) and complex **2** (b). The TGA plot of complex **1** (c) and complex **2** (d)

At the aim of detecting products’ phase purity, the investigations of powder X-ray diffraction on the complexes synthesized was performed (Figure 3(a)). For simulated PXRD fashions, its peak positions are in conformity with the outcomes of the experiment, and this displays that the crystallographic architecture genuinely represents the products of bulk crystal. The crystal samples preferred selection may be the cause of the strength differences. TGA was performed to investigate the thermal behaviors for the estimation of the as-created two compounds’ thermal stabilities at 10 °C·min^−1^ heating rate under the atmosphere of N_2_. As reflected in the Figure 3(c), the complex **1**’s TGA curve reveals 6.27% of weightlessness (from 27 to 323°C), which equivalent to the release of 2 lattice and 4 coordinated molecules of water (with the calculated value of 6.35%). After 323°C, the second weightlessness was appeared, which suggests that the entire skeleton collapses owing the organic ligands decomposition. For the complex **2**, between 27 and 265°C, there is 3.24% of weightlessness, which is associated with the removal of a lattice free molecule of water (with 3.76% calculated value). Subsequently, the complex **2**’s skeleton starts to collapse (Figure 3(d)).

### Magnetic behavior

**Figure 4.**
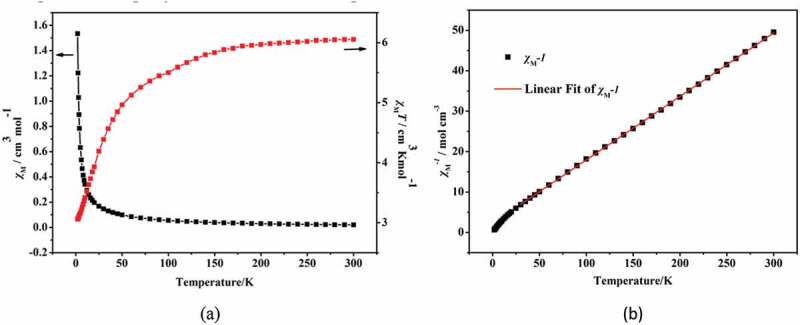
The temperature dependence of *χ*_*M*_ and and *χ_M_T* for the complex **1** between 2 and 300 K (a); The 1/*χ*_*M*_ plot for **1** in 25–300 K (b). The red line indicates the best fit of Curie-Weiss

The two compounds’ different temperature-dependent magnetic performances were detected in length under 1 kOe applied field from 2 K to 300 K. As revealed in the Figure 4(a), at 300 K, the value of *χ_m_T* for the complex **1** is 6.05 cm^3^·K·mol^−1^, this value is slightly higher than the value of pure spin value for 3 separated Co^2+^ ion (5.625 cm^3^ K mol^−1^) (*S* = 3/2), this phenomenon indicates an important orbital contribution of the Co^2+^ ions with high-spin value [18,19]. At 2 K, with the decrease of temperature, the *χ_m_T* value reduced gradually to the minimum value (which is 3 cm^3^ K mol^−1^), exhibiting antiferromagnetic phenomenon in the complex **1**. Moreover, between 25 K and 300 K, the complex **1**’s reciprocal susceptibility (1/*χ*_*M*_) conform to the law of Curie–Weiss [*χ*_*M*_ = *C*/(*T*−*θ*)], in which the values of Weiss constant *θ* and Curie constant *C,* respectively, are −14.2 K and 6.4 cm^3^·mol^−1^·K (Figure 4(b)). The negative value of *θ* exhibits the antiferromagnetic phenomenon in complex **1** and even through the contribution of Co(II) spin-orbit coupling effect also exists in complex **1** [20].

**Figure 5.**
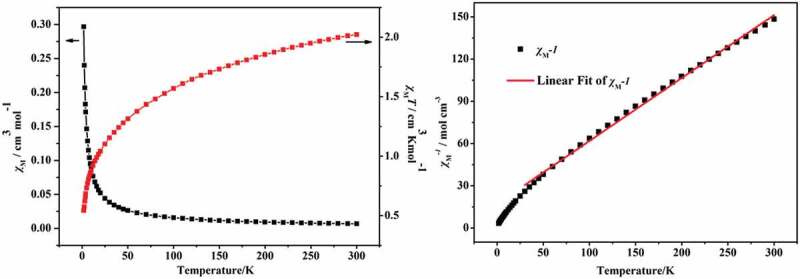
The temperature dependence of *χ*_*M*_ and *χ_M_T* for the complex **2** from 2 K to 300 K (a); The 1/*χ*_*M*_ plot for **2** in 25–300 K (b). The red line indicates the best fit of Curie-Weiss

The structure analysis shows that the center Co(II) ions in **1** and **2** show the similar coordination configuration and the adjacent Co(II) ions in two compounds are bound by the organic ligands, thus the two compounds show the similar magnetic properties. The investigation of the magnetic properties for **2** has also been explored, which show the similar magnetic behavior to **1** (Figure 5(a)). The complex **2**’s variable-temperature magnetic performances were determined in length under 1 kOe applied field between 2 K and 300 K. At 300 K, the experimented value of *χ_m_T* is 2.0 cm^3^·K·mol^−1^, this value is greater than the expected value of high pure spin for a separated Co(II) ions (1.875 cm^3^·K·mol^−1^) (*g* = 2 and *S* = 3/2). When the temperature reduced to 2 K, the value of *χ_m_T* reduces gradually to the minimum value (which is 0.54 cm^3^·K·mol^−1^), indicating that the complex **2** also exists antiferromagnetic coupling. The reciprocal susceptibility (1/*χ*_*M*_) temperature dependence also conform to the law of Curie–Weiss: *χ*_*M*_ = *C*/(*T*−*θ*) from 25 K to 300 K, where the values of Weiss constant *θ* and Curie constant *C,* respectively, are −38.9 K and 2.3 cm^3^·mol^−1^·K (Figure 5(b)). The negative *θ* suggests the existence of antiferromagnetic phenomenon in complex **2**.

### Inhibitory activity of the compound on the pro-inflammatory cytokines content in hematoma capsule

**Figure 6.**
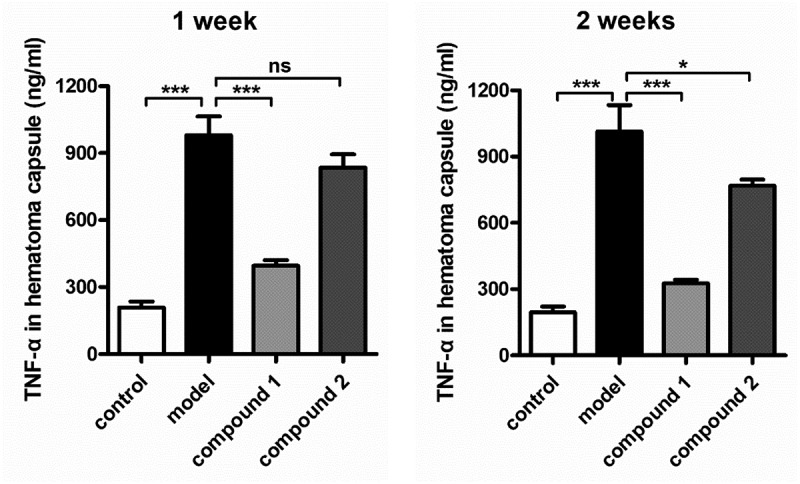
Compound **1** decreased the pro-inflammatory cytokines content in the hematoma capsule. Intracranial subdural space was injected with the autologous venous blood every two 3-day intervals, the injection of compounds were carried out for the treatment (at 2.5 μg/kg concentration). The enzyme-linked immunosorbent assay detection kit was employed for the pro-inflammatory cytokines determination in the hematoma capsule

After creating the novel compounds having fresh structure, the compounds’ influence against the content of pro-inflammatory cytokines in hematoma capsule was measured firstly. As a result, the ELISA detection kit of TNF-α was implemented in the present work. As the outcomes displayed in the Figure 6, we can observe that in model group, the TNF-α levels was much higher, in contrast to the level in control group, with the P value less than 0.005. After the treatment of compound **1**, the TNF-α levels in hematoma capsule were decreased remarkably. The compound **1**’s inhibition was much stronger than the inhibition of **2**.

### Promotion activity of the compound on anti-inflammatory cytokines content in hematoma capsule

**Figure 7.**
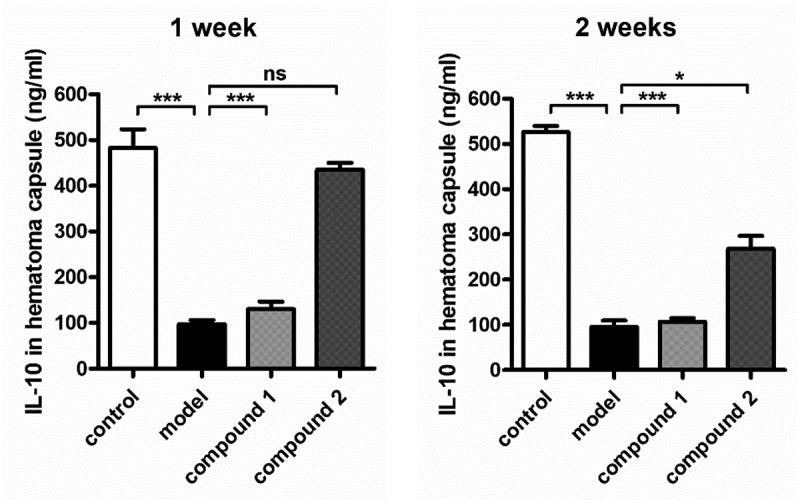
Compound enhanced the levels of anti-inflammatory cytokines in the hematoma capsule. Intracranial subdural space was injected with the autologous venous blood every two 3-day intervals, the injection of compounds were performed for the treatment (with 2.5 μg/kg concentration). In the hematoma capsule, the content of IL-10 was determined via applying the enzyme-linked immunosorbent assay detection kit

As former exhibited, the compound **1** revealed outstanding inhibitory activity against the pro-inflammatory cytokines content in the hematoma capsule, this effect is stronger than the **2**. Moreover, we also investigated the compounds’ influence against the anti-inflammatory cytokines content in the hematoma capsule. The data in the Figure 7 indicated that in model group, the level of IL-10 in the hematoma capsule was obviously lower, in comparison with the level in control group. After treatment of compound **1**, in the hematoma capsule, the level of IL-10 was evidently enhanced. Nevertheless, the **2** only exhibited little effect against the content of IL-10.

### No cytotoxicity of compounds 1 and 2 showed on the normal human cells

**Figure 8.**
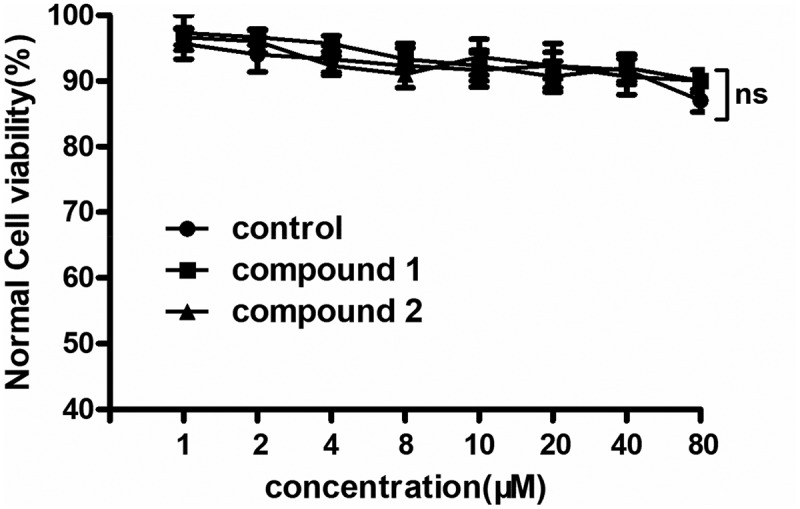
Both compounds **1** and **2** has no cytotoxicity on the normal human cells. The normal human cells in the logical growth phage were collected and seeded into the 96 well plates. Then, compounds **1** and **2** was added for treatment with serial different dilutions (1, 2, 4, 8, 10, 20, 40 and 80 μM). The viability of the normal human cells was determined with CCK-8 detection

In the above experiments, compound **1** was much stronger than compound **2** on reducing the pro-inflammatory cytokines content and up-regulating the anti-inflammatory cytokines content in the hematoma capsule. However, for the further application of the new compound, the cytotoxicity of compounds **1** and **2** on the normal human cells was still need to be further explored. Thus, the CCK-8 assay was conducted in this present research. The results of the CCK-8 suggested that both compounds **1** and **2** has no inhibitory effect on the viability of the normal human cells. There were no significant differences between those group, with P > 0.05. This data indicated both compounds **1** and **2** has no cytotoxicity (Figure 8).

## Conclusion

To sum up, we have produced two CPs based on Co(II) triumphantly by utilizing the mixed-ligand synthesis method. The results of structural characterization suggest that the **1** possesses a two-dimensional layered net where the ligand of opda^2−^ reveals the trans-conformation and as the bridging building block, it extends a one-dimensional (Co–mbib) polymer chain containing loop into a two-dimensional net. In complex **2**, the ligands of mpda^2−^ reveal the cis-conformation, and it can be regarded as the building block with ‘V’-type, connecting the Co(II) atoms with the ligand of mbib to produce a one-dimensional chain containing loop. The two compounds’ magnetic performances suggest that between the adjacent metal ions, there present the antiferromagnetic coupling. The outcomes revealed that the **1** was more outstanding than the **2** against the treatment of CSDH through balancing the pro-inflammatory cytokines and anti-inflammatory cytokines. In summary, the compound could significantly reduce the pro-inflammatory cytokines level and increase the anti-inflammatory cytokines levels. Compared with complex **2**, the complex **1**’s biological effect was much stronger. The CCK-8 assay indicated that both compounds **1** and **2** showed no cytotoxicity on the normal human cells.
